# The Impact of Non-pharmacological Interventions Measures Against COVID-19 on Respiratory Virus in Preschool Children in Henan, China

**DOI:** 10.1007/s44197-023-00168-3

**Published:** 2023-12-04

**Authors:** Hui Yan, Bo Zhai, Fang Yang, Penggao Wang, Yang Zhou

**Affiliations:** Henan Provincial Clinical Research Center for Pediatric Diseases, Henan Key Laboratory of Pediatric Genetics and Metabolic Diseases, Children’s Hospital Affiliated to Zhengzhou University, Henan Children’s Hospital, Zhengzhou Children’s Hospital, Zhengzhou, 450018 China

**Keywords:** Respiratory virus, COVID-19, Pandemic, Preschool children, Henan

## Abstract

**Objectives:**

To investigate the long-term effects of non-pharmacological interventions (NPIs) measures on the epidemiological characteristics of common respiratory viruses in preschool children in Henan, China.

**Methods:**

This was a retrospective observational study containing data from 17 prefecture-level cities in Henan, China. We analyzed and compared laboratory results and clinical data of preschool children presenting to outpatient clinics for acute respiratory infections (ARTI) after COVID-19 (January 2020–October 2022) and before COVID-19 (December 2017–December 2019). Each year was divided into quarters. The ratio of the odds ratios (*OR*s) of testing positive for eight respiratory viruses in each year after the pandemic to the prepandemic period was estimated applying a generalized linear model (GLM), using the mean of the positive detection rates in 2018–2019 as a reference.

**Results:**

A total of 11,400 children were enrolled from December 2017 to October 2022. The number of positive detections for all respiratory viruses decreased in 2020–2022 compared to the average of 2018–2019. Human respiratory syncytial virus (hRSV), human rhinovirus (hRV), and influenza virus (IFV) accounted for a larger proportion of all detected viruses before COVID-19 pandemic, whereas hRV, human bocavirus (hBoV), and human adenovirus (hAdV) accounted for a significantly larger proportion after COVID-19 pandemic. The positive detection rates of enveloped viruses [IFV, human parainfluenza virus (hPIV), hRSV, human metapneumovirus (hMPV), and human coronavirus (hCoV)] decreased sharply and the seasonal activity of these viruses was weakened, while the positive detection rates of non-enveloped viruses (hRV, hBoV, and hAdV) increased, especially hRV. The conditions described above tended to occur more frequently in boys and children older than 1 year, and they were also more sensitive to the NPIs.

**Conclusions:**

NPIs transformed the epidemiological profile of common respiratory viruses among preschool children during the COVID-19 pandemic. To improve the overall public health response to all respiratory viruses, interventions targeting non-enveloped viruses need to be strengthened to mitigate their continued transmission.

## Introduction

Respiratory viral infections, caused by dozens of various types of viruses, represent the most common cause of childhood morbidity and mortality as well as a global health concern [1–3]. Viruses had been reported to account for up to 90% of the causes of pneumonia in infants under 1 year old and about 50% in preschool children [4]. The common viruses causing ARTI in children include viruses with an envelope structure, such as IFV, hPIV, hRSV, hMPV, and hCoV, as well as viruses without envelope structure, including hRV, hBoV, and hAdV [5]. Since severe acute respiratory syndrome coronavirus 2 (SARS-CoV-2) was first identified in 2020, NPIs, including social distancing, wearing masks, hand hygiene, limiting crowd gathering, and restricting outdoor activities, had been implemented worldwide to control the spread of COVID-19. Some studies had shown that these measures also altered the prevalence of other respiratory viral infections in adults and children, but these findings were inconsistent [6–10]. In contrast, there remains a lack of systematic studies on whether and how NPIs affect other respiratory pathogens in preschool children, one of the vulnerable groups. Hence, analysis of the distribution and epidemiological changes of respiratory viruses in preschool children before and after the COVID-19 pandemic is essential for the prevention and control of respiratory infections in children.

We conducted a retrospective study of the results of eight respiratory viruses commonly detected in preschool children (under 7 years of age) in Henan Province, China. We analyzed the respiratory virus data of the last 5 years (December 2017–October 2022) to obtain the changes on the number of positives, positivity rates, population gender, age, virus type, and epidemic season in various respiratory virus before and after the epidemic to better understand the impact of COVID-19 pandemic and NPIs on respiratory virus infections in children.

## Methods

### Study Design

We obtained the data from the Children's Hospital Affiliated to Zhengzhou University, a tertiary‐level children's health care institution integrating medical care, health care, public health, teaching, research, and training, and one of the largest children's specialty hospitals in Henan Province, central China. The children participating in the study were all from 17 prefecture-level cities in Henan Province, and all had lived there for more than 6 months.

In this study, we randomly selected preschool children who were seen in outpatient clinics for ARTI from December 2017 to October 2022 as study subjects. According to the Centers for Disease Control and Prevention(CDC) criteria, enrolled children met the following criteria: (i) children aged younger than 7 years, regardless of gender; (ii) confirmed fever (temperature > 37.5 °C); (iii) one or more respiratory symptoms and/or signs within 14 days of onset [cough, sore throat, sputum, shortness of breath, lung auscultation abnormality (rale or wheeze), tachypnea, and chest pain] [11]; (iv) complete pathogenetic test data were available for each specimen. The exclusion criteria for this study were (i) children with nonviral respiratory infections; (ii) children with recurrent visits within 1 week; (iii) children with hospital-acquired infections; (iv) children with congenital pulmonary airway malformations and compromised immune systems; (v) children infected with COVID-19.

Study subjects were grouped: (i) by year; (ii) by gender; (iii) by age, that was, infancy (≤ 1 year), early childhood (1 to 3 years), and preschool (3 to 7 years). The study was approved by the Ethics Committee of Children's Hospital Affiliated to Zhengzhou University. This retrospective study did not involve any personal private information of the patients and only statistical analysis of the available test data was performed. The committee waived the need for individual informed consent.

### Data Collection and Specimen Detection

We obtained the demographic information of enrolled children from their electronic medical records. Respiratory specimens (including nasal swabs, pharyngeal swabs, nasopharyngeal aspirates or sputum, and bronchoalveolar lavage fluid) were obtained from all enrolled children immediately after admission by trained staff following standard operating procedures. And the specimens were immediately (within 12 h) transferred to the clinical laboratory for respiratory virus testing via sterile virus transfer media (Beijing Yongkang Biotechnology Co., Ltd., China). The respiratory specimens were kept at a temperature of 2–8 °C at all times during preservation and transportation for testing.

Upon arrival at the laboratory, clinical specimens were processed in a Class II biosafety cabinet. The supernatant was then centrifuged to obtain a supernatant for screening of multiple respiratory pathogens. The total nucleic acids of each specimen were extracted using EasyPure Viral DNA/RNA Kit (TransGen Biotech, Beijing, China) in accordance with the manufacturer's instructions. IFV, hPIV, hRSV, hMPV, hCoV, and hRV were detected by RT-PCR, and hAdV and hBoV were tested by PCR according to the standard operating protocol (SOP) of surveillance developed by China CDC. If either of the target viruses was detected in the sample, the patient was considered positive for that virus. In cases where two viruses were detected in the same clinical sample, the respective viruses were counted separately.

### Statistical Analysis

The number of positive infections for each respiratory virus in different years and seasons was expressed as an absolute number, and the positive detection rate was expressed as a percentage. We plotted the time-series changes of eight respiratory viruses during 2018–2022, describing the changes in the number of positive respiratory virus infections, positive detection rates, and virus distribution in children across years and seasons. A Chi-square test or Fisher's exact test was used to compare the differences in the positive rates of various viruses before the COVID-19 pandemic (2018–2019) and after the COVID-19 pandemic (2020–2022) (using the mean of virus positivity rates in 2018–2019 compared with the mean of 2020–2022). The ratio of the odds ratios (*ORs*) of testing positive for eight respiratory viruses in each year after the pandemic (2020–2022) to the pre-pandemic period 2018–2019 was estimated applying a generalized linear model (GLM), using the mean of the positive detection rates in 2018–2019 as a reference. Relevant confounding factors were also controlled for, including child gender, age, health status, and residence; parental age, education level, occupation, and monthly household income. Analysis was also performed by gender and different age groups. When analyzing by gender stratification, no adjustment was made for the child's gender; when analyzing by age group stratification, no adjustment was made for the child's age. Statistical significance was evaluated with two-sided *p* values at the level of *α* = 5%. All statistical analyses were conducted in the IBM SPSS Statistics for Windows (version 27.0.; IBM Corp.) and R software (version 4.0.3, R Development Core Team 2020).

## Results

### Clinical Characteristics of Included Children

A total of 11,400 children with respiratory infections were included in this study. Overall, 5774 (50.6%) of the 11,400 specimens tested positive for at least one of the eight viruses, and the virus detection rate increased after the epidemic, with statistically significant differences in virus detection rates between years (*P* < 0.001) (Table 1).

**Table 1:** Characteristics of included children from 2018 to 2022 in Henan, China

	2018 (*n* = 4010)	2019 (*n* = 4510)	2020 (*n* = 790)	2021 (*n* = 950)	2022 (*n* = 1140)	*X*^*2*^	*P*	Total (*N* = 11,400)
*Age*								
≤ 1 year	1960 (48.9%)	2232 (49.5%)	484 (61.3%)	627 (66.0%)	680 (59.6%)	456.000	** < 0.001**	5983 (52.5%)
1 ~ 3 years	1243 (31.0%)	1489 (33.0%)	237 (30.0%)	256 (27.0%)	285 (25.0%)			3510 (30.8%)
3 ~ 7 years	807 (20.1%)	789 (17.5%)	69 (8.7%)	67 (7.0%)	175 (15.4%)			1907 (16.7%)
*Gender*								
Male	2113 (52.7%)	2336 (51.8%)	420 (53.2%)	499 (52.5%)	602 (52.8%)	5.437	0.626	5970 (52.4%)
Female	1897 (47.3%)	2174 (48.2%)	370 (46.8%)	451 (47.5%)	538 (47.2%)			5430 (47.6%)
*Region*								
Urban	2606 (65.0%)	3075 (68.2%)	628 (79.5%)	762 (80.2%)	898 (78.8%)	100.203	**0.026**	7969 (70.0%)
Rural	1404 (35.0%)	1435 (31.8%)	162 (20.5%)	188 (19.8%)	242 (21.2%)			3431 (30.0%)
*Child health*								
Good or very good	3413 (85.1%)	3824 (84.8%)	683 (86.5%)	813 (85.6%)	980 (86.0%)	312.000	** < 0.001**	9713 (85.2%)
Fair or poor	597 (14.9%)	686 (15.2%)	107 (13.5%)	137 (14.4%)	160 (14.0%)			1687 (14.8%)
*Virus detection rate*	1941 (48.4%)	2253 (50.0%)	475 (60.1%)	525 (55.3%)	580 (50.9%)	526.016	** < 0.001**	5774 (50.6%)

### Comparison of Respiratory Virus Positivity Rates by Year

**Fig. 1:**
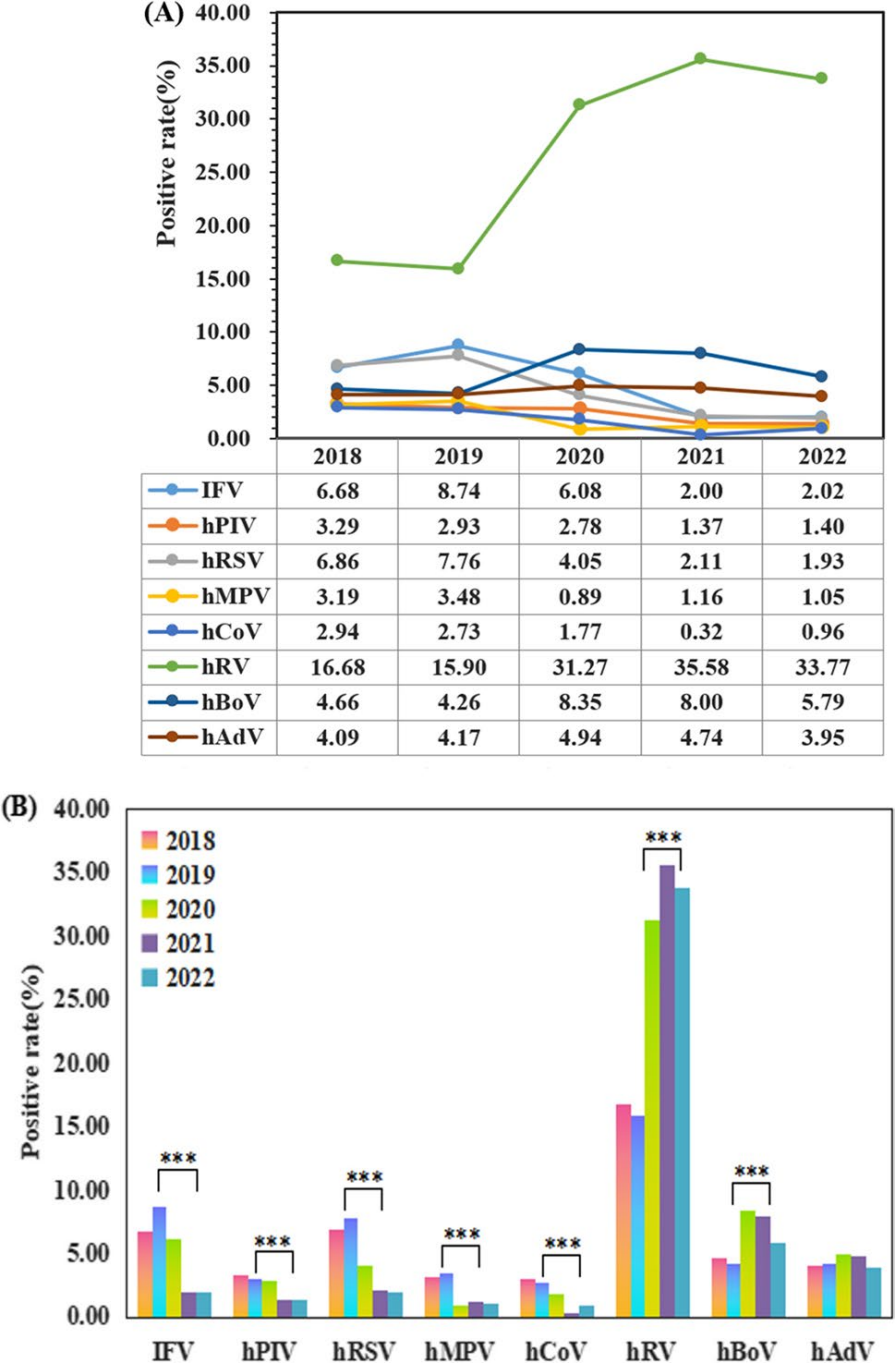
Changes in the positive rates of eight respiratory viruses in preschool children from 2018 to 2022 in Henan, China. **A** Trends in eight respiratory viruses positivity from 2018 to 2022 in Henan, China; **B** comparison of the positive rates of eight respiratory viruses before and after the COVID-19 pandemic in Henan, China. ***The *P* value is less than 0.001

We investigated eight respiratory viruses (IFV, hPIV, hRSV, hMPV, hCoV, hRV, hBoV, and hAdV) in children with respiratory infections. As shown in Fig. 1, the mean detection rate of IFV was 7.77% in 2018–2019, which was higher than in 2020–2022 (3.13%) after the COVID-19 epidemic, with a statistically significant difference (*P* < 0.001). The positive detection rate of hPIV showed a decreasing trend year by year, and the mean detection rate of hPIV was higher between 2018 and 2019 (3.10%) than between 2020 and 2022 (1.77%), with a statistically significant difference (*P* < 0.001). The mean detection rate of hRSV was higher between 2018 and 2019 (7.34%) than between 2020 and 2022 (2.57%), with a statistically significant difference (*P* < 0.001). The mean detection rate for hMPV was 3.35% in 2018–2019, which was higher than in 2020–2022 (1.04%), with a statistically significant difference (*P* < 0.001). The mean detection rate for hCoV was higher between 2018 and 2019 (2.83%) than between 2020 and 2022 (0.97%), with a statistically significant difference (*P* < 0.001).

In addition, the mean detection rate of hRV was 16.27% in 2018–2019, which was lower than in 2020–2022 (33.68%) after the COVID-19 epidemic, with a statistically significant difference (*P* < 0.001). The mean detection rate for hBoV was lower between 2018 and 2019 (4.45%) than between 2020 and 2022 (7.22%), with a statistically significant difference (*P* < 0.001). The mean detection rate of hAdV was slightly lower between 2018 and 2019 (4.13%) than between 2020 and 2022 (4.48%), with no statistically significant difference (*P* = 0.537) (Fig. 1).

### Changes in the Epidemic Season of Respiratory Virus

**Fig. 2:**
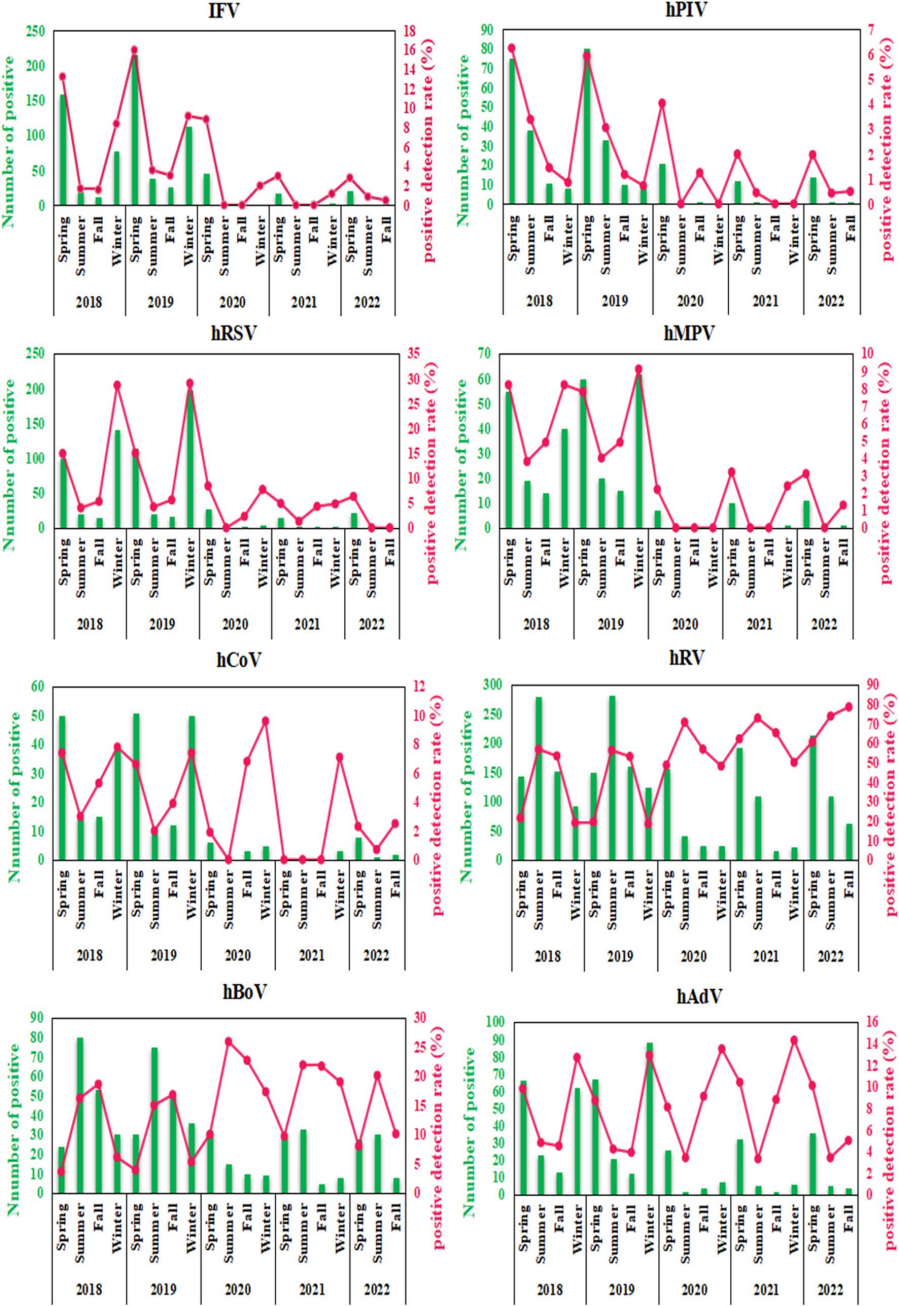
The number of positive infections and positive detection rates of eight respiratory viruses in preschool children from 2018 to 2022 in Henan, China

The results showed a reduction in the number of positive infections for each virus in 2020–2022 compared to 2018 and 2019, with the least in 2020. The positive detection rates of IFV, hPIV, hRSV, hMPV, and hCoV showed a decreasing trend, while the positive detection rates of hBoV and hAdV did not decrease and the positive detection rate of hRV increased. In addition, all respiratory virus infections in preschool children were distinctly seasonal before the COVID-19 pandemic, with IFV, hPIV, and hRSV infections prevalent in spring, hRV and hBoV infections in summer and fall, and IFV, hRSV, hMPV, hCoV, and hAdV infections in winter, whereas the seasonality of viral infections was diluted during the COVID-19 pandemic (Fig. 2).

### Changes in the Proportion of Respiratory Viruses

**Fig. 3:**
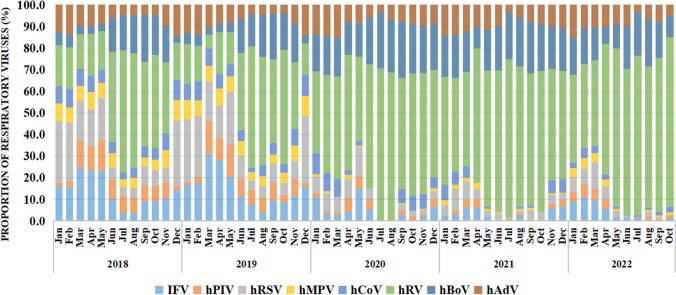
Proportion of respiratory viruses detected in preschool children from 2018 to 2022 in Henan, China

Further, we analyzed the distribution of respiratory viruses among preschool children before and after the COVID-19 pandemic. The results revealed an unusual change in the proportion of respiratory viruses detected since the COVID-19 outbreak. The detection rates and distribution of respiratory viruses were affected by COVID-19 pandemic, and after the first wave of the COVID-19 outbreak (after January 2020), although only few enveloped viruses (IFV, hPIV, hRSV, hMPV, and hCoV) were detected, more non-enveloped viruses (hRV, hBoV, hAdV) were still detected in children. There was also some variation in the proportion of viruses. hRSV, hRV, and IFV accounted for a larger proportion of all detected viruses before COVID-19 pandemic, whereas hRV, hBoV, and hAdV accounted for a significantly larger proportion after COVID-19 pandemic (Fig. 3).

### Effects of NPIs on Activity of Respiratory Viruses

The pattern of the phase-specific ORs (Table 2) was further revealed largely consistent with that of the percentage changes in Figs. 1 and 2, with some subtle differences. Without stratification, the ORs of enveloped viruses (IFPV, hPIV, hRSV, hMPV, and hCoV) were less than 1 in 2020, 2021, and 2022, and the implementation of NPIs reduced the positive detection rates of these viruses. The ORs of non-enveloped viruses (hRV, hBoV, and hAdV) were all greater than 1, and NPIs had little protective effect on these viruses, especially hRV. After stratification by gender, the same effect was still observed in boys, and only the ORs of both hRSV and hRV were statistically significant over 3 years in girls, with diminished hRSV activity and enhanced hRV activity. After stratification by age group, it was still observed that the ORs of IFV, hPIV, hRSV, hMPV, and hCoV were all less than 1 and the ORs of hRV, hBoV, and hAdV were all greater than 1 in each age group after the COVID-19 epidemic due to the implementation of NPIs measures. Most of these ORs were statistically significant in children older than 1 year, whereas these effects were not significant in children younger than 1 year.

**Table 2:**
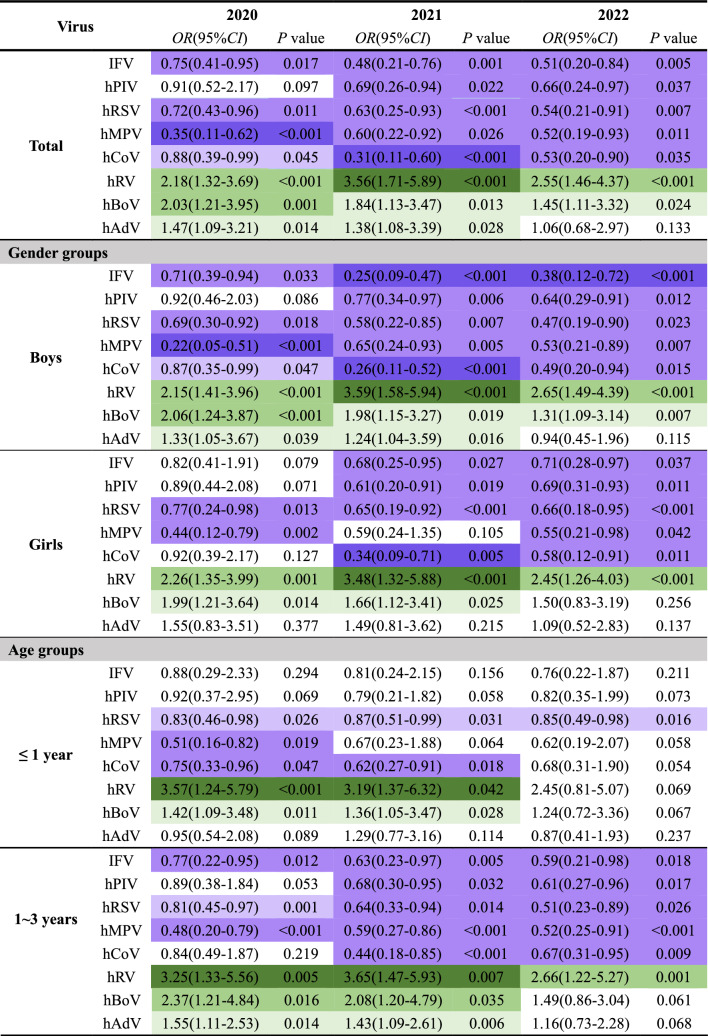

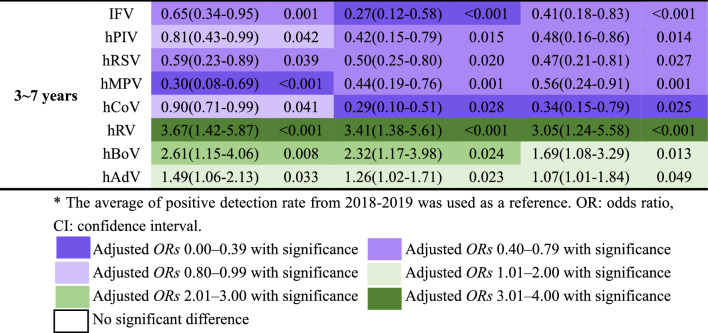
GLM-estimated the odds ratios (*ORs*) for the odds of a positive test for eight respiratory viruses in each year after the pandemic (2020 to 2022) compared to within 2018–2019 before the pandemic in Henan, China

## Discussion

In this study, we retrospectively assessed the positive detection rates for eight respiratory viruses commonly seen in children with ARTI attending before and after the COVID-19 epidemic. The overall positive detection rate was 48.4% in 2018, 50.0% in 2019, 60.1% in 2020, 55.3% in 2021, and 50.9% in 2022, which was lower than the positive detection results reported in the other studies (62.4 ~ 85.2%) [12–15]. The number of specimens tested in 2020–2022 decreased compared with 2018–2019, but the overall positive rate increased, probably because the series of NPIs against SARS-CoV-2 also effectively stopped the transmission of other respiratory viruses. However, these measures had increased the resistance to visit the hospital, and only those children with more severe symptoms and signs tended to come to the hospital. Therefore, the number of infections decreased without a decrease in positive detection rates.

Our results demonstrated that NPIs seemed to be more effective against enveloped viruses, such as IFV, hPIV, hRSV, hMPV, and hCoV than non-enveloped viruses, such as hRV, hBoV, and hAdV. And these NPIs measures did not even have any effect on reducing the transmission of non-enveloped viruses, which was also consistent with the results of some studies [16–18]. This may be mainly attributed to the superior thermal properties of non-enveloped viruses, their ability to tolerate dry and acidic environments, and their relatively low sensitivity to alcohol, which allows them to survive longer in external environments and remains active and infectious under unfavorable conditions [19, 20]. Previous studies have shown that masks offer less protection against hRV infection than against IFV or hCoV [21, 22]. However, our findings indicated that NPIs’ measures against SARS-CoV-2 could not eliminate not only hRV but also non-enveloped viruses, such as hBoV and hAdV.

Our data also revealed that infants under 3 years old were more susceptible to respiratory viral infections than children 3–7 years old, mainly due to physiological differences in the respiratory tract and immaturity of the immune system [23, 24]. And infants under 3 years old performed worse on NPIs measures than older children, so the study data presented that infants were less sensitive to the protective effect of NPIs measures. In terms of gender, our study found that boys were more susceptible to respiratory viruses and more sensitive to the protective effects of NPIs measures. Probably mainly because boys preferred outdoor activities and socialization compared to girls [25, 26], and the implementation of NPIs measures reduced these activities. Further studies were also required to fully elucidate the mechanisms of sexual differences in respiratory viral infections.

There were also some limitations in our study: a causal relationship between viral activity and NPIs measures could not be inferred from the data obtained. Our analysis was based on the rate of positive tests, for which changes in health care delivery practices had little effect on the results, but it still could not be excluded that the pandemic had changed the profile of children seeking medical assistance for ARTI. Finally, we conducted this study at a single institution. Nevertheless, the institution where the specimens were collected was the core pediatric hospital in the region. Therefore, it is considered to be a strong reflection of the prevalence of respiratory viruses in preschool children in the region and the results obtained are of some informative value.

## Conclusion

In conclusion, NPIs altered the proportion of respiratory viruses detected in preschool children and diminished the rates of infection with enveloped viruses but not with non-enveloped viruses during the COVID-19 pandemic. To improve the global public health response to all respiratory transmitted viruses, interventions targeting non-enveloped viruses need to be strengthened to mitigate their continued transmission. The educational aims of this study are to have informed clinical healthcare professionals about the impact of NPIs on the pattern of respiratory viral infections in children, and to provide basic research data for the integration of resources in healthcare organizations after the epidemic. It also provides theoretical basis and practical reference for guardians on how to prevent respiratory viral infections in preschool children. In addition, future research directions should consider how the epidemiologic characteristics of respiratory viral infections in preschool children change after the elimination of NPIs, and whether enveloped viral infections will make a comeback. And continuous and dynamic surveillance of respiratory data will help prevent major outbreaks of respiratory viral infections after the epidemic.

## Data Availability

The data included in this study were collected by the researchers and were in principle not publicly available unless specifically needed.

## Abbreviations

NPIs: Non-pharmacological interventions
ARTI: Acute respiratory infections
ORs: Odds ratios
GLM: Generalized linear model
IFV: Influenza virus
hPIV: Human parainfluenza virus
hRSV: Human respiratory syncytial virus
hMPV: Human metapneumo virus
hCoV: Human coronavirus
hRV: Human rhinovirus
hBoV: Human bocavirus
hAdV: Human adenovirus
SARS-CoV-2: Severe acute respiratory syndrome coronavirus 2
CDC: Centers for Disease Control and Prevention
SOP: Standard operating protocol
CI: Confidence interval
